# Evaluation of clinical response according to category and level of evidence for therapeutic plasma exchange indications: A Single-Center Experience

**DOI:** 10.12669/pjms.41.9.12597

**Published:** 2025-09

**Authors:** Ali Dogan, Gokhan Aydeniz

**Affiliations:** 1 Ali Dogan Van Yuzuncu Yil University, Faculty of Medicine, Hematology Department, Van, Turkiye; 2 Gokhan Aydeniz Van Yuzuncu Yil University, Faculty of Medicine, Hematology Department, Van, Turkiye

**Keywords:** Category, Evidence-based medicine, Plasma exchange, Treatment outcome

## Abstract

**Objective:**

The present study investigates the indications of patients who underwent therapeutic plasma exchange (TPE) based on the category and level of evidence outlined in the 2023 guidelines of the American Society for Apheresis (ASFA) and evaluates their treatment responses.

**Methodology:**

This retrospective study, the records of patients who underwent TPE at Van Yüzüncü Yıl University Medical Faculty Hospital between January 2020 to December 2024 were reviewed, including their demographic characteristics, indications, plasmapheresis procedure data and complications. The indications were classified according to the categories, levels of evidence and treatment responses laid out in the ASFA 2023 guidelines.

**Results:**

A total of 234 adult patients were included in the study, of whom 56.4% were female. Hematological (41.9%) and neurological (38.5%) disorders accounted for the majority of the conditions treated with TPE. Of the total, 53% of the patients were classified as Category-I, while the total proportion of patients with Grade-IA, IB and IC evidence levels was 64.5%. The complete response rate was 35.5% in Category-I patients, the partial response rate was 48.6% in Category-II patients and the no response rate was 50% in Category-III patients. Patients with hematological and neurological disorders recorded high clinical response rates, while rarer conditions showed low clinical response rates. A significant relationship was found between category and level of evidence and clinical response (p<0.05).

**Conclusion:**

The clinical efficacy of TPE is significantly associated with the ASFA category and the level of evidence of the indications. The findings of the present study highlight the need to refer to evidence-based guidelines when planning TPE procedures.

## INTRODUCTION

Therapeutic plasma exchange (TPE) is an extracorporeal treatment approach in which plasma is separated from the blood to facilitate the delivery of targeted therapeutic interventions in specific diseases.^1^,^2^ After blood is withdrawn from the body and processed extracorporeally to separate the plasma - the liquid component - from the other elements using various techniques, the remaining blood components are combined with appropriate replacement fluids and returned to the patient.^3^ This procedure eliminates the risk associated with harmful substances in the plasma, including autoantibodies, immune complexes, paraproteins, toxins and pathogenic metabolites.^4^,^5^

The American Society for Apheresis (ASFA) regularly publishes guidelines defining indications for therapeutic apheresis (TA) for the standardization of clinical practice. The ASFA Guidelines (updated in 2023) provide a scientific framework of the categories and levels of evidence for TA applications, thereby aiding clinicians in decision-making processes.^6^ This guideline classifies TA indications into Categories: I, II, III and IV and provides detailed evidence levels for each category (Grade-IA, IB, IC, IIA, IIB, IIC). Previous studies in the literature report that the clinical efficacy of TPE is increased in diseases with higher categories and levels of evidence.^6^,^7^ The present study classifies the indications of patients undergoing TPE based on the categories and levels of evidence outlined in the ASFA 2023 guidelines and investigates the concordance of their treatment responses with the guidelines.

## METHODOLOGY

This retrospective, single-center study was carried out based on a review of the data of patients who underwent TPE at Van Yuzuncu Yil University Medical Faculty Hospital between January 2020 and December 2024.

### Ethical approval:

The study was granted approval by the Ethics Committee of our university (No:2025/01-44; Date: February 4, 2025).

### Patient Selection:

Data were collected from the accessible records of 263 patients who underwent TPE and 29 patients who did not meet the inclusion criteria were subsequently excluded from the study. A total of 234 adult patients were thus included in the study. For each patient, the findings of the first plasmapheresis period were analyzed.

### Inclusion Criteria:

- Aged 18 years or older, TPE indication corresponding to the conditions specified in the ASFA 2023 Guidelines and complete and accurate clinical, laboratory and complication data accessible from patient records.

### Exclusion Criteria:

- Insufficient treatment process and response evaluation data, presence of contraindications for plasmapheresis and non-compliance with treatment.

### Data Collection:

TPE procedural data, such as the number of sessions, treatment frequency, types of replacement fluids used and plasma volumes processed, were obtained from the records of the therapeutic apheresis center. Demographic data such as age and gender, TPE indications, comorbidities, laboratory findings and treatment responses were obtained from the hospital information system and patient records. The patients’ TPE indications were classified based on the categories and levels of evidence laid out in the ASFA 2023 guidelines and the consistency of their treatment responses with these classifications was analyzed. Categorization of indications according to the ASFA 2023 guidelines: Category-I: Conditions for which TA is considered a first-line treatment, either alone or in combination with other therapies. Category-II: Conditions for which TA is considered a second-line treatment, either alone or in combination with other therapies. Category-III: Conditions for which TA is considered potentially beneficial, although existing evidence is limited. Category-IV: Limited data regarding the efficacy of TA. Evidence: Grade-II evidence: Strong suggestion. Grade-II evidence: Poor recommendation Evidence quality A: High-quality evidence. Evidence quality B: Medium-quality evidence. Evidence quality C: Poor-quality or very low-quality evidence.

### Evaluation of Treatment Responses: Complete Response:

Defined as the full resolution of disease symptoms, accompanied by normalization of laboratory tests and clinical findings. Partial Response: Defined as a partial improvement in disease symptoms, along with a partial resolution of clinical findings and laboratory test results. Non-response: Defined as the absence of any significant improvement in disease symptoms, clinical findings or laboratory test results.

### Statistical Analysis:

Continuous variables were expressed as median values (minimum-maximum), while categorical variables were expressed as frequency and percentage (%) values. TPE indications were classified into three categories (Category-I, II, III) and six levels of evidence (Grade-IA, IB, IC, IIA, IIB, IIC), in line with the ASFA 2023 guidelines. Fisher’s exact test could not be applied to all subgroups due to the large cell counts between groups and the low frequencies in certain subgroups. Response status was thus reclassified into two groups for the assessment of statistical significance between categories, as “response present” (complete response + partial response) and “no response”. Fisher’s exact test was used for Category-I and Category-III indications, while Pearson’s Chi-square test was applied for Category-II. A p-value of less than 0.05 was considered statistically significant. The analyses were performed using SPSS (Version 22.0. IBM Corp., Armonk, NY).

## RESULTS

The study included 234 patients, 56.4% of whom were women. The median age of the patients was 38 years (range: 18-88). TPE was performed for the various indication groups across seven different medical specialties. The majority of patients were classified under Category-I at a rate of 53% (n=124) and under evidence levels Grade-IA, IB and IC at a rate of 64.5% (n=151). The demographic and clinical characteristics of the patients are summarized in Table-I.

**Table-I:** Demographic and clinical characteristics of patients undergoing plasmapheresis.

Clinical parameters	n (%) or median (min-max)
Age, years	38 (18-88)
***Gender***	
Male	102 (43,6)
Female	132 (56,4)
***Indication group***	
Hematological diseases	98 (41,9)
Neurological diseases	90 (38,5)
Endocrinological diseases	20 (8,5)
Kidney diseases	15 (6,4)
Intensive care diseases	6 (2,6)
Rheumatological diseases	4 (1,7)
Dermatological diseases	1 (0,4)
***Category***	
Category-I	124 (53)
Category-II	72 (30,8)
Category-III	38 (16,2)
***Evidence (Grade)***	
Grade-IA-IB-IC	151 (64,5)
Grade IIA-IIB-IIC	83 (35,5)
***Additional disease***	
Yes	117 (50)
No	117 (50)
Plasma volume ratio (1:1 or 1:2)	1 (1-2)
Duration of procedure, days	5 (1-51)
Number of procedures	5 (1-55)
***Frequency of procedures***	
Daily	131 (56)
Every other day and other	103 (44)
***Anticoagulant***	
Heparin	129 (55,1)
Asit sitrat dextroz-A	105 (44,9)
***Replacement fluid***	
FFP	178 (76)
Albumin	56 (24)
***Complication***	
None	213 (91)
Allergic reaction	12 (5,1)
Catheter dysfunction	5 (2,1)
Hypocalcemia	2 (0,9)
Hypotension	2 (0,9)
***Treatment response***	
Complete response	69 (29,5)
Partial response	83 (35,5)
No response	82 (35)

FFP: Fresh frozen plasma.

**Fig.1:**
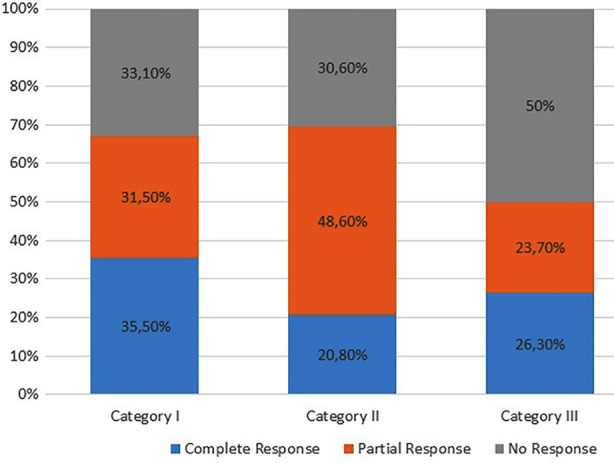
Response rates of patients according to category levels.

TPE was administered for a total of 23 different indications. The distribution of diseases and their corresponding categories and levels of evidence according to the ASFA 2023 guidelines are shown in Table-II. In addition, the clinical responses of these diseases to treatment are presented in Table-III. The diseases for which TPE was most frequently performed were multiple sclerosis (MS) (17.9%; n=42) and thrombotic thrombocytopenic purpura (TTP) (15.4%; n=36), respectively. When the overall treatment response was assessed, complete response was observed in 29.5% of patients (n=69), partial response in 35.5% (n=83) and non-response in 35% (n=82). The response rates of patients are shown graphically according to category levels (Fig.1).

**Table-II:** Categories of indications for plasmapheresis and levels of evidence according to ASFA 2023 guidelines.

Disease/condition	n (%)	Category	Grade
Multiple sclerosis	42 (17,9)	II	1A
Thrombotic microangiopathy, thrombotic thrombocytopenic purpura	36 (15,4)	I	1A
AIDP	24 (10,3)	I	1A
Thrombotic microangiopathy, complement mediated	21 (9)	I- III	IIC
Hyperviscosity in hypergammaglobulinemia	18 (7,7)	I	IB
Myasthenia gravis	17 (7,3)	I	IB
Thrombotic microangiopathy, infection mediated	15 (6,4)	III	IIC
Thyroid storm	15 (6,4)	II	IIC
Thrombotic microangiopathy, coagulation mediated	7 (3)	III	IIC
ANCA Associated RPGN	6 (2,6)	III	IIC
Sepsis (multiorgan failure)	6 (2,6)	III	IIA
Renal transplantation rejection	6 (2,6)	I	IB
Hyperlipidemia	5 (2,1)	II	IB
Optic neuritis	4 (1,7)	II	IB
Polyarteritis nodosa	2 (0,9)	II	IIC
Limbic encephalitis	2 (0,9)	III	IIC
Focal segmental glomerulosclerosis	2 (0,9)	II	IIC
Henoch-schonlein purpura	1 (0,4)	III	IIC
Catastrophic antiphospholipid syndrome	1 (0,4)	I	IIC
Goodpasture syndrome	1 (0,4)	I	IC
Acute disseminated encephalomyelitis	1 (0,4)	II	IIC
Systemic lupus erythematosus	1 (0,4)	II	IIC
Pemphigus vulgaris	1 (0,4)	III	IIB
Total	234 (100)		

AIDP: Acute inflammatory demyelinating polyradiculoneuropathy; ANCA: Anti-Neutrophil Cytoplasmic Antibody; RPGN: Rapidly Progressive Glomerulonephritis.

**Table-III:** Clinical responses to treatment of plasmapheresis indications according to ASFA 2023 guidelines.

Disease/condition	Treatment response		
	Complete response n (%)	Partial response n (%)	No response n (%)
Multiple sclerosis	3 (7,1)	23 (54,8)	16 (38,1)
Thrombotic microangiopathy, thrombotic thrombocytopenic purpura	27 (75)	4 (11,1)	5 (13,9)
AIDP	3 (12,5)	14 (58,3)	7 (29,2)
Thrombotic microangiopathy, complement mediated	5 (23,8)	6 (28,6)	10 (47,6)
Hyperviscosity in hypergammaglobulinemia	6 (33,3)	10(55,6)	2 (11,1)
Myasthenia gravis	3 (17,6)	5 (29,4)	9 (52,9)
Thrombotic microangiopathy, infection mediated	10 (66,7)	3 (20)	2 (13,3)
Thyroid storm	9 (60)	6 (40)	0 (0)
Thrombotic microangiopathy, coagulation mediated	0 (0)	1 (14,3)	6 (85,7)
ANCA Associated RPGN	0 (0)	3 (50)	3 (50)
Sepsis (multiorgan failure)	0 (0)	0 (0)	6 (100)
Renal transplantation rejection	0 (0)	0 (0)	6 (100)
Hyperlipidemia	3 (60)	1 (20)	1 (20)
Optic neuritis	0 (0)	1 (25)	3 (75)
Polyarteritis nodosa	0 (0)	2 (100)	0 (0)
Limbic encephalitis	0 (0)	1 (50)	1 (50)
Focal segmental glomerulosclerosis	0 (0)	1 (50)	1 (50)
Henoch-schonlein purpura	0 (0)	1 (100)	0 (0)
Catastrophic antiphospholipid syndrome	0 (0)	0 (0)	1 (100)
Goodpasture syndrome	0 (0)	0 (0)	1 (100)
Acute disseminated encephalomyelitis	0 (0)	0 (0)	1 (100)
Systemic lupus erythematosus	0 (0)	1 (100)	0 (0)
Pemphigus vulgaris	0 (0)	0 (0)	1 (100)
Total	69(29,5)	83 (35,5)	82 (35)

The statistical analyses revealed both the categories and levels of evidence to be significantly associated with response rates within their respective groups (Table-IV). The complete response rate was highest in Category-I (35.5%, p=0.005), whereas the partial response rate was significantly higher in Category-II (48.6%, p=0.042). The non-response rate (50%) was significantly high in Category-III and the heterogeneity observed at a subgroup level was also found to be statistically significant (p=0.008).

**Table-IV:** Response distribution and statistical analysis of patients according to category and evidence level.

Category	Grade	Response present		No response n (%)	Total n (%)	p value
		Complete response n (%)	Partial response n (%)			
I	1A	30 (50)	18 (30)	12 (20)	60 (100)	0,005
	IB	9 (22)	15 (36,6)	17 (41,5)	41 (100)	
	IC	0 (0)	0 (0)	1 (100)	1 (100)	
	IIC	5 (22,7)	6 (27,3)	11 (50)	22 (100)	
	Total	44 (35,5)	39 (31,5)	41 (33,1)	124 (100)	
II	1A	3 (7,1)	23 (54,8)	16 (38,1)	42 (100)	0,042
	IB	3 (33,3)	2 (22,2)	4 (44,4)	9 (100)	
	IIC	9 (42,9)	10 (47,6)	2 (9,5)	21 (100)	
	Total	15 (20,8)	35 (48,6)	22 (30,6)	72 (100)	
III	IIA	0 (0)	0 (0)	6 (100)	6 (100)	0,008
	IIB	0 (0)	0 (0)	1 (100)	1 (100)	
	IIC	10 (32,3)	9 (29,0)	12 (38,7)	31 (100)	
	Total	10 (26,3)	9 (23,7)	19 (50)	38 (100)	

Category 1, Fisher’s exact test; Category 2, Pearson chi-square; Category 3, Fisher’s exact test.

## DISCUSSION

In the present study, the indications of patients who underwent TPE are classified according to the ASFA 2023 guidelines and the corresponding treatment responses are analyzed. The majority of patients in the study had hematological and neurological conditions, for which TPE is expected to provide clinical benefit and the observed distribution of indications was consistent with existing literature.^2^,^6^ The majority of patients in the study were classified as Category-I, which was the category that showed the highest rate of clinical response. This finding serves as strong support for the recognition of conditions classified as Category-I in the ASFA 2023 guidelines as primary indications for TPE.^6^ TTP, classified under Category-I, was identified as the most common indication for TPE among the hematological conditions analyzed, with a complete response rate of 75%, consistent with the 70-80% range reported in previous studies investigating the efficacy of TPE in TTP.^8^,^9^

MS was the most common neurological disease for which TPE was administered in the present study. Patients in the study were administered TPE in line with the recommendations in the literature for severe MS episodes that are unresponsive to high-dose corticosteroid therapy at disease onset and for relapsing cases.^10^ The partial response rate among MS patients in the present study was 54.8%, suggesting that TPE primarily leads to symptomatic improvement in MS, while cases of complete clinical remission are rare.

A high clinical response was observed in patients diagnosed with acute inflammatory demyelinating polyneuropathy (AIDP) who underwent TPE in the present study, supporting the efficacy of TPE as a first-line treatment option during the acute phase of AIDP.^11^ The high rate of partial responses in the present study demonstrates the contribution of plasmapheresis to the process of functional recovery. In contrast, a large majority of the patients diagnosed with myasthenia gravis (MG) who underwent TPE in the present study did not achieve any clinical response. The low response rate observed in patients with MG may be attributed to both the timing of the initiation of plasmapheresis and the influence of accompanying comorbidities. Consistent with previous studies, the present study reveals TPE to be an effective therapeutic option for patients with MG, particularly during disease episodes marked by respiratory failure and bulbar symptoms.^12^

No therapeutic response could be achieved in approximately half of the patients in the present study who received TPE with a diagnosis of complement-mediated thrombotic microangiopathy (CM-TMA). Our findings support those of the study by Khandelwal et al. revealing TPE to be an effective treatment modality for patients with CM-TMA and supporting its use as a bridging therapy during the transition to eculizumab therapy.^13^

In our study, all patients who developed hyperviscosity syndrome due to monoclonal gammopathy were diagnosed with multiple myeloma. Clinical response was achieved in the acute phase in the majority of these patients who underwent TPE. Concurring with the findings of the study by Dhakal et al., the present study further demonstrates the suitability of TPE for the management of the neurological and vascular complications associated with hyperviscosity in patients with multiple myeloma.^14^

Clinical response was achieved in the vast majority of patients treated with TPE in the present study following a diagnosis of infection-associated thrombotic microangiopathy (Hemolytic Uremic Syndrome - HUS). It is believed that the high response rate may be attributable to the effective implementation of concurrent supportive therapies. Menne et al. reported that TPE provides limited clinical benefit to patients with HUS, but may be considered as a supportive therapy, particularly in cases with severe hemolysis and organ dysfunction.^15^

In the present study, all patients who underwent TPE due to thyroid storm demonstrated a clinical response. In a study of patients who underwent TPE due to hyperthyroidism, Keklik M et al. reported a clinical response rate of 91%.^16^ The ASFA 2023 guidelines recommend TPE in cases of thyroid storm for patients who do not respond to first-line therapies or to encourage clinical stabilization prior to surgery.^6^ The indications for TPE in the present study were consistent with the guidelines and are in line with the existing literature.

The clinical conditions for which TPE was administered in the present study, including coagulation-mediated thrombotic microangiopathy, ANCA-associated rapidly progressive glomerulonephritis (RPGN), sepsis-related multiorgan dysfunction, renal transplant rejection, catastrophic antiphospholipid syndrome, Goodpasture syndrome, limbic encephalitis, acute disseminated encephalomyelitis, focal segmental glomerulosclerosis, Henoch-Schönlein purpura and pemphigus vulgaris, are rare indications with differing pathophysiologies and limited levels of supporting evidence according to ASFA guidelines.^6^

In our study, both the low number of cases with such indications and the limited clinical response rates to TPE highlight the need for a critical reassessment of the clinical efficacy of TPE in these settings, particularly in cases of sepsis.^6^,^17^ A low complication rate was noted in the present study, with allergic reactions being the most frequently reported. In parallel with our study, Kazmi et al. reported a 12% complication rate in a large patient population undergoing TPE.^18^ Review by Weinstein R., a 20-25% decrease in plasma ionized calcium is expected during plasmapheresis.^19^ The low rate of hypocalcemia observed in the present study may be attributed to the preferential use of heparin as an anticoagulant and the administration of 1,554 mg calcium infusion during the procedure in patients receiving acid citrate dextrose solutions, formula A (ACD-A).

### Strengths:

This study include the high number of patients and the evaluation of a wide spectrum of indications from multiple branches of medicine. Furthermore, its categorization of indications according to the ASFA 2023 guidelines and its comparison of treatment responses against these categories and levels of evidence provide the study with an evidence-based framework that supports further assessment.

### Limitations:

The retrospective design of the study may have resulted in information gaps during the data collection process and may have introduced potential subjective biases in the evaluation of treatment responses. In addition, the small number of patients with certain rare indications, particularly those classified under Category-III, may have reduced the statistical power of the subgroup analyses. Therefore, prospective and multicenter studies are particularly needed for rare indications.

## CONCLUSION

The administration of TPE in cases with Category-I indications resulted in a high rate of clinical response, while partial response rates were higher for Category-II indications and no response rates were higher for Category-III indications. The findings of our study suggest that TPE is appropriate as the primary treatment approach in cases of TTP and thyroid storm. The present study has shown that the effectiveness of TPE applications is influenced not only by the disease category, but also the level of evidence. It can thus be concluded that assessing TPE applications based on the indication categories and evidence levels set out in the ASFA 2023 guidelines can play a key role in predicting treatment success.
